# Evaluation of Plant Phenolic Metabolites as a Source of Alzheimer's Drug Leads

**DOI:** 10.1155/2014/843263

**Published:** 2014-05-29

**Authors:** Yara Hassaan, Heba Handoussa, Ahmed H. El-Khatib, Michael W. Linscheid, Nesrine El Sayed, Nahla Ayoub

**Affiliations:** ^1^Department of Pharmaceutical Biology, Faculty of Pharmacy and Biotechnology, German University in Cairo, Cairo 11835, Egypt; ^2^Laboratory of Analytical and Environmental Chemistry, Department of Chemistry, Humboldt-Universität zu Berlin, 12489 Berlin, Germany; ^3^Department of Pharmacology and Toxicology, Faculty of Pharmacy, Cairo University, Cairo 11562, Egypt; ^4^Department of Pharmacology and Toxicology, Faculty of Pharmacy & Biotechnology, German University in Cairo, Cairo 11835, Egypt; ^5^Department of Pharmaceutical Biology, Faculty of Pharmacy, Ain-Shams University, Cairo 11613, Egypt; ^6^Department of Pharmaceutical Biology, Faculty of Pharmacy, British University in Cairo, Cairo 11613, Egypt

## Abstract

Epidemiological studies have proven an association between consumption of polyphenols and prevention of Alzheimer's disease, the most common form of dementia characterized by extracellular deposition of amyloid beta plaques. The aim of this study is pharmacological screening of the aqueous alcohol extract of *Markhamia platycalyx* leaves, *Schotia brachypetala* leaves and stalks, and piceatannol compared to aqueous alcohol extract of *Camellia sinensis* leaves as potential Alzheimer's disease drugs. LC-HRESI(-ve)-MS^n^ was performed to identify phenolics' profile of *Schotia brachypetala* stalks aqueous alcohol extract and revealed ten phenolic compounds as first report: daidzein, naringin, procyanidin isomers, procyanidin dimer gallate, quercetin 3-*O*-rhamnoside, quercetin 3-*O*-glucuronide, quercetin hexose gallic acid, quercetin hexose protocatechuic acid, and ellagic acid. Alzheimer's disease was induced by a single intraperitoneal injection of LPS. Adult male Swiss albino mice were divided into groups of 8–10 mice each receiving treatment for six days. *In vivo* behavioral tests (Y maze and object recognition) and *in vitro* estimation of amyloid beta 42 by ELISA showed significant differences between results of treated and nontreated animals.

## 1. Introduction

**Figure 1:**
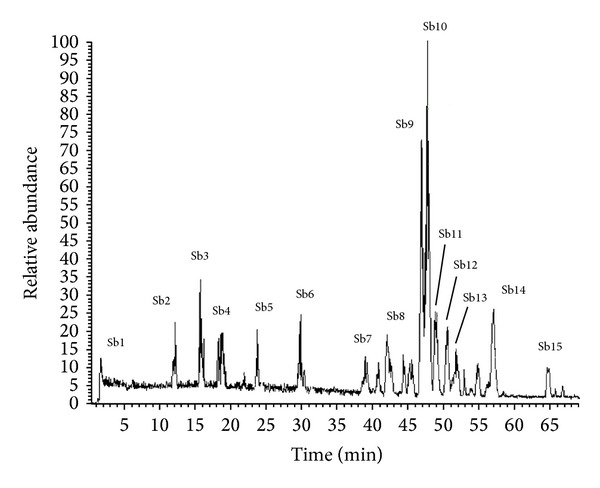
LC-MS of aqueous alcohol* Schotia brachypetala* stalk extract.

Alzheimer's disease (AD) is the most prevalent neurodegenerative disease in the elderly population, which eventually leads to complete incapacity and death of patients. It is characterized by the progressive and selective loss of neurons and synapses, extracellular deposition of amyloid beta plaques, and formation of intracellular neurofibrillary tangles composed of hyperphosphorylated tau protein mainly in the brain regions that are involved in cognitive processes [1]. Natural occurring dietary polyphenolics have received considerable recent attention as alternative candidates for Alzheimer's disease therapy [2]. Epidemiological studies have proven an association between the consumption of polyphenolic rich foods or beverages and the prevention of neurological diseases including Alzheimer's disease [2]. The ability of polyphenols to cross the intestinal wall of mammals [3] and the ability of some to cross the blood brain barrier [4] confers their biological properties. Moreover, one of the major properties of this group of compounds is their ability to interact with peptides and proteins; such interaction can prove valuable at the biological level in general and especially in Alzheimer's disease [5]. Green tea EGCG inhibits *β*-amyloid induced memory dysfunction evaluated by passive avoidance and water maze tests [6]. Piceatannol showed neuroprotective effect against toxicity induced by amyloid beta peptides in rat hippocampal cell cultures. Also decreased amyloid beta 1–42 induced fluorescence in a TH-t fluorescence assay [7]. Bark extracts of* Schotia brachypetala* exhibited pronounced monoamine oxidase-B inhibition activity [8] and monoamine oxidase inhibitors are useful in treating AD [9]. Phenolic glycosides from* Markhamia stipulata *were isolated, mainly being verbascosides [10]. Verbascosides are phenylpropanoids that have cytoprotective action connected to their antioxidant and chelating capacity [11]. This data suggests genus* Markhamia* can be used to treat oxidative stress related neurodegenerative disease as AD. Thus the current study was performed with the main aim of pharmacological screening of piceatannol and the phenolic rich extracts of leaves of* Camellia sinensis, Markhamia platycalyx, *and* Schotia brachypetala *and stalks of* Schotia brachypetala* using behavioural mouse models (y maze, object recognition) as promising Alzheimer's disease drug leads and using* Camellia sinensis* as reference standard. Further pharmacological screening of extracts was performed by mouse amyloid beta 42 ELISA assay. Characterization of phenolics' profiling in stalks of* Schotia brachypetala* aqueous alcohol extract was executed by LC-MS and X calibur software (Figure 1).

## 2. Materials and Methods 

### 2.1. Plant Material

Aerial parts of* Markhamia platycalyx, Camellia sinensis*, and* Schotia brachypetala* and stalks of* Schotia brachypetala *were collected from the Orman Garden, Giza, Egypt, in May 2012. The authenticity of species was confirmed by Professor Dr. Mohamed El Gebaly, Professor of Taxonomy at the National Research Center, Egypt. Voucher specimen was deposited at the herbarium of the Faculty of Pharmacy and Biotechnology, Department of Pharmaceutical Biology, German University in Cairo, Egypt.

### 2.2. Extraction

The leaves of* Markhamia platycalyx, Camellia sinensis, *and* Schotia brachypetala *(500 g) and stalk of* Schotia brachypetala* (500 g) [12] were exhaustively extracted with distilled water (5 L), individually. The extract was evaporated in* vacuo* at low temperature till dryness to yield (12 g) for each of* Markhamia platycalyx, Camellia sinensis*, and* Schotia brachypetala* leaves and (15 g) of* Schotia brachypetala *stalks. Then, the dry solid residues obtained from evaporation of aqueous extracts were extracted with ethanol. The dried aqueous extract of each plant was dissolved in ethanol, individually. This step was followed by evaporation of the filtrate to obtain crude phenolic content, 7 g from leaves of* Markhamia platycalyx*,* Camellia sinensis*, and* Schotia brachypetala *and 5 g from* Schotia brachypetala* stalks. The dry residual powder of aqueous ethanol extract was kept in tightly closed sample tubes at room temperature.

### 2.3. LC-HRESI-MS-MS

LC-HRESI-MS-MS was performed on a Bruker micro-TOF-Q Daltonics (API) Time-of-Flight mass spectrometer (Bremen, Germany), coupled to 1200 series HPLC system (Agilent Technologies, Waldbronn, Germany), equipped with a high performance autosampler, binary pump, and PDA detector G 1314 C (SL). Chromatographic separation was performed on a Superspher 100 RP-18 (75 × 4 mm i.d.; 4 *μ*m) column (Merck, Darmstadt, Germany).

### 2.4. Identification of Phenolic Compounds of Aqueous Ethanol Extract of* Schotia brachypetala* Stalk by LC-HRESI-MS-MS

The solvents were (A) 2% acetic acid (pH 2.6) and (B) 80% methanol, 2% acetic acid, and pH 2.6. The gradient elution was from 5% to 50% B at 30°C at a flow rate of 100 *μ*L/min. The ionization technique was an ion spray (pneumatically assisted electrospray). The mass spectrometer was operated in the negative mode. Negative mode conditions were applied to the instrument as follows: capillary voltage, 4000 V; end plate offset, −500 V; heated dry nitrogen gas temperature, 200°C; at flow rate 10 L/min, the gas flow to the nebulizer was set at pressure 1.6 bar. For collision-induced dissociation (CID) MS-MS measurements, the voltage over the collision cell varied from 20 to 70 eV. Argon was used as collision gas. Data analysis software was used for data interpretation. Sodium formate was used for calibration at the end of LC-MS run. Interpretation for ESI-MS was performed by Xcalibur 2.1 software from Thermo Scientific (Berlin, Germany).

### 2.5. Animals

Adult male Swiss albino mice weighing from 20 to 30 g each were used. They were brought from the National Institute of Research in Egypt. All animals were housed in alternating 12 h dark/night cycles at suitable room temperature one week before experimental intervention. They had unrestricted access to food and water throughout the period of investigation. All experiments were performed under the anesthesia with 2% isoflurane to minimize the suffering. Animal procedures were approved by the Ethics Committee at the German University in Cairo in agreement with recommendations of the National Institute of Health Guide for Care and Use of Laboratory Animals.

### 2.6. Experimental Protocol

Alzheimer's disease was induced by intraperitoneal injections of Lipopolysaccharide (0.8 mg/kg) once [19]. Green tea extract from leaves of* Camellia sinensis *was used as the reference standard. Doses and vehicles used to dissolve drugs were chosen based on the literature. The extracts and piceatannol were dissolved in DMSO and then completed to final volume with saline [20]. The mice were divided into groups of 8–10 mice each receiving i.p. injections after being weighed. LPS group (Group I) received 0.1 mL of 0.8 mg/kg LPS i.p. injections once [19]. The normal group (Group II) received 0.1 mL DMSO or saline i.p. injections once. The green tea treated group (Group III) received 0.8 mg/kg LPS i.p injection once followed by 100 mg/kg/day green tea extract from* Camellia sinensis* [21] for 6 days. The* Schotia brachypetala* leaves treated group (Group IV) received 0.8 mg/kg LPS i.p. injection once followed by 100 mg/kg/day [22]* Schotia brachypetala* leaves extract for 6 days. The* Schotia brachypetala* stalk treated group (Group V) received 0.8 mg/kg LPS i.p. injection once followed by 100 mg/kg/day [22]* Schotia brachypetala *stalk extract for 6 days.* Markhamia platycalyx *leaves treated group (Group VI) received 0.8 mg/kg LPS i.p. injection once followed by 100 mg/kg/day [23]* Markhamia platycalyx *extract for 6 days. Piceatannol treated group (Group VII) received 0.8 mg/kg LPS i.p injection once followed by 2.5 mg/kg/day [6] piceatannol for 6 days. Y maze and object recognition were performed as* in vivo* tests on the 7th day to evaluate the extracts and piceatannol's anti-AD potential and confirmed by* ex vivo* tests. After the* in vivo* tests, the mice brains were harvested. Each brain was divided into 2 hemispheres and frozen at −80 degrees Celsius to be later homogenized and used for* ex vivo* ELISA analysis.

### 2.7. Object Recognition Test

Object recognition test was performed in accordance with the study done by [24]. The natural propensity of animals to explore novel objects more relative to familiar ones is used as an index of stimulus recognition. The test involves a “sample” phase, in which a to-be-remembered object is encountered, followed by a delay and then a test phase in which the sample object is presented together with a novel object. Discrimination ratio is the ratio of attempts towards the new object against the old one. Exploratory attempt is defined as the animal directing its nose towards the object at a distance of 2 cm or less. Climbing on object was not considered an exploration. Increase in discrimination ratio is indicative that the mouse is exploring the new object more.

It was calculated by the following equation:

(1) Discrimination  ratio  =Number  of  attempts  of  animal  to  novel  objectTotal  number  of  attempts  of  the  animal  to  both  objects

see [25].

### 2.8. Y Maze Test

Y maze was carried out in accordance with the study done by [26]. An animal must remember which arm it had entered on a previous occasion to enable it to alternate its choice on a following trial. In recent years, spontaneous alternation behaviour has been enthusiastically embraced by behavioral pharmacologists and others as a quick and relatively simple test of memory [27]. The test is carried out in Y maze shaped apparatus with three arms. Each arm is labeled either A, B, or C. The test consists of two phases, with the first being the training phase. The mouse is allowed to move freely through the maze for 8 minutes. 24 hours later, the mouse is allowed to move for 8 minutes and its movements are recorded. Every time the mouse enters an arm, with all of its limbs inside, its letter is written down. The number of alternations means the successive entries into three different arms in overlapping triplet sets [28] (e.g., ABCBACA = 3). Total arm entries are simply the total number of arms entered (e.g., ABCBACA = 7). The percentage alternation was calculated according to [28] using the following formula:

(2) $$\begin{array}{c} \frac{\text{number}\;\text{of}\;\text{alternations}}{\;\left(\text{Total}\;\text{arm}\;\text{Entries}-2\right)}\times 100. \end{array}$$

### 2.9. Tissue Sampling

After performing the behavioural tests, each brain was split into 2 hemispheres and placed in an eppendorf tube and weighed and then stored at −80°C. The brain was used later for homogenization using appropriate volume of PBS/protease inhibitor and guanidine/tris HCl solution to bring final concentration of guanidine to 5 M. This solution was added in aliquots of 100 *μ*L while grinding the hemisphere. The homogenates were mixed for 3-4 hours at room temperature. At this stage, the samples were stable and can be freeze-thawed many times. They were frozen until assay of amyloid beta 42 levels with ELISA (Invitrogen, USA) [29].

### 2.10. ELISA and Measurement of Amyloid Beta 42

Samples were centrifuged at 16,000 g for 20 min at 4°C. Supernatant was diluted to bring the final guanidine concentration to 0.1 M using Dulbecco's phosphate buffered saline with 5% BSA and 0.03% Tween-20. The supernatant was stored on ice until assay of amyloid beta 42 levels. The Mouse A*β*42 levels were assayed with solid phase sandwich enzyme linked immunosorbent assay (ELISA) according to manufacturer's instructions [29].

### 2.11. Statistical Analysis

Statistical analysis was performed using instant automated software (Graph Pad Prism software version 5.01, Inc., San Diego, California, USA). Results were expressed as mean ± standard error of mean (SEM). Results from behavioural tests were analyzed using unpaired *t*-test to compare the two selected groups of Y maze or object recognition test. The level of significance was set at *P* = 0.05 with *P* < 0.05 indicating significant change. The confidence interval was fixed at 95%. Graphical representation of results was conducted using Graph Pad Prism 5 software.

## 3. Results

### 3.1. *In Vivo* Results

#### 3.1.1. Effects of Lipopolysaccharide,* Camellia sinensis*, Piceatannol,* Markhamia platycalyx, *and* Schotia brachypetala* Leaves and* Schotia brachypetala* Stalk on the Mean Alternation Percentage Using Y Maze Test

**Figure 2:**
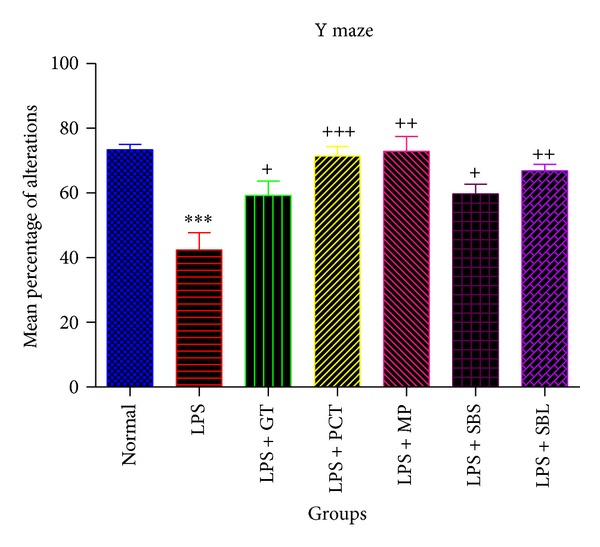
Effects of Lipopolysaccharide, green tea, piceatannol,* Markhamia platycalyx, *and* Schotia brachypetala* leaves and* Schotia brachypetala* stalks on mean alternation percentage using Y maze test. Normal group animals were injected with DMSO or saline 0.9% i.p. once with nonsignificant results between them. The 6 other groups had Alzheimer's disease that was induced by i.p. injection of LPS once. Five of these groups were treated with either PCT (2.5 mg/kg), or 100 mg/kg of GT, MP, SBS, or SBL daily for 6 days. The animals of each group (*n* = 8–10) were subjected to Y maze testing on the 7th day and the sequence of arm entries was recorded for 8 minutes for every mouse. Then the percentage alternations were calculated for each mouse. Statistical analysis was performed using unpaired *t*-test to compare every two groups. Each value represents mean ± standard error of mean. *Significantly different from normal at *P* < 0.05.**Significantly different from normal at *P* < 0.01. ***Significantly different from normal at *P* < 0.001. ^+^Significantly different from LPS at *P* < 0.05. ^++^Significantly different from LPS at *P* < 0.01. ^+++^Significantly different from LPS at *P* < 0.001. MPA: mean alternation percentage. DMSO: dimethyl sulfoxide. LPS: Lipopolysaccharide. GT: green tea extract of* Camellia sinensis.* PCT: piceatannol. SBS:* Schotia brachypetala* leaves. SBL:* Schotia brachypetala* stalk. MP:* Markhamia platycalyx*. i.p. intraperitoneal injection.

Administration of LPS (0.8 mg/kg i.p) then on the 7th day subjecting mice to Y maze showed a significant decrease in the mean percentage alternations by 42.25% compared to normal group. The mean percentage alternations were significantly increased in animals that were treated with piceatannol (2.5 mg/kg/day),* Camellia sinensis* leaves (100 mg/kg/day),* Markhamia platycalyx* leaves (100 mg/kg/day),* Schotia brachypetala* leaves (100 mg/kg/day), and* Schotia brachypetala* stalk (100 mg/kg/day) for 6 days by 68.78%, 39.74%, 72.14%, 40.9%, and 57.85%, respectively, compared to LPS group (Figure 2).

#### 3.1.2. Effects of Lipopolysaccharide,* Camellia sinensis*, Piceatannol,* Markhamia platycalyx, *and* Schotia brachypetala* Leaves and* Schotia brachypetala* Stalks on the Mean Discrimination Ratio Using Object Recognition Test

Administration of 0.8 mg/kg LPS and then subjecting animals to object recognition test on 7th day showed significant decrease in mean discrimination ratio by 46.16% when compared to the normal group.

**Figure 3:**
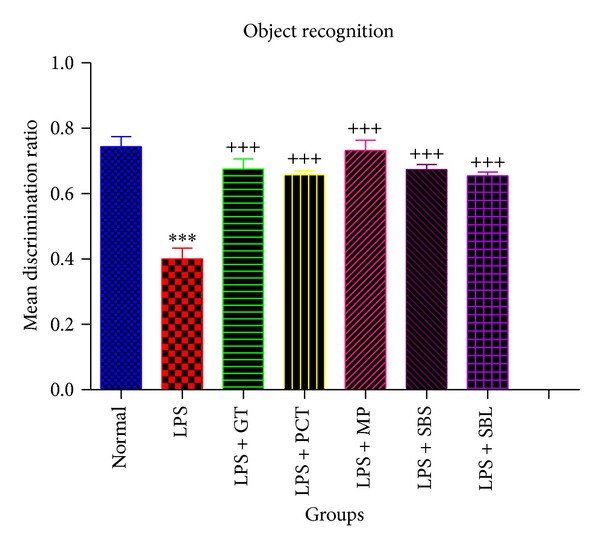
Effects of Lipopolysaccharide, green tea, piceatannol,* Markhamia platycalyx, *and* Schotia brachypetala* leaves and* Schotia brachypetala* stalk on the mean discrimination ratio using object recognition test. Normal group animals were injected with DMSO or saline 0.9% i.p. once with nonsignificant results between them. The 6 other groups had Alzheimer's disease that was induced by i.p. injection of LPS once. Five of these groups were treated with either PCT (2.5 mg/kg) or 100 mg/kg of GT, MP, SBS, or SBL daily for 6 days. The animals of each group (*n* = 8–10) were subjected to object recognition testing on the 7th day testing and exploration attempts towards each object within 4 minutes were recorded for each mouse. Then the discrimination ratio was calculated for each mouse. Statistical analysis was performed using unpaired *t*-test to compare every two groups. Each value represents mean ± standard error of mean. *Significantly different from normal at *P* < 0.05. **Significantly different from normal at *P* < 0.01. ***Significantly different from normal at *P* < 0.001. ^+^Significantly different from LPS at *P* < 0.05. ^++^Significantly different from LPS at *P* < 0.01. ^+++^Significantly different from LPS at *P* < 0.001. MDR: mean discrimination ratio. DMSO: dimethyl sulfoxide. LPS: Lipopolysaccharide. GT: green tea extract of* Camellia sinensis*. PCT: piceatannol. SBS:* Schotia brachypetala* leaves. SBL:* Schotia brachypetala* stalk. MP:* Markhamia platycalyx*. i.p. intraperitoneal injection.

Discrimination ratio was significantly increased in animals that were treated with piceatannol (2.5 mg/kg/day),* Camellia sinensis *(100 mg/kg/day),* Markhamia platycalyx *(100 mg/kg/day), and* Schotia brachypetala* leaves (100 mg/kg/day) and* Schotia brachypetala *stalk (100 mg/kg/day) for 6 days of treatment by 64.5%, 68.75%, 83%, 68.25 %, and 65% respectively, when compared with LPS group (Figure 3).

### 3.2. *Ex Vivo* Results

#### 3.2.1. Effects of Lipopolysaccharide,* Camellia sinensis*, Piceatannol,* Markhamia platycalyx*, and* Schotia brachypetala* Leaves and* Schotia brachypetala* Stalk on the Mean Mouse Amyloid Beta 42 Concentration Using ELISA Assay

Mean amyloid beta 42 present in mouse brains was evaluated by mouse A*β*42 ELISA kit. LPS group showed significantly higher concentration of amyloid beta when compared to normal group by almost 2-fold.

**Figure 4:**
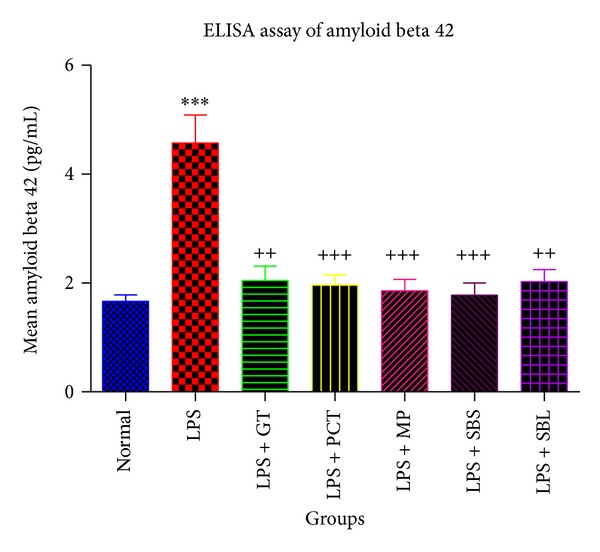
Effects of Lipopolysaccharide, green tea, piceatannol,* Markhamia platycalyx, *and* Schotia brachypetala* leaves and* Schotia brachypetala* stalk on mean mouse amyloid beta 42 concentration using ELISA assay. The normal group was injected with saline 0.9% or DMSO i.p. once with nonsignificant results between them. All the other groups were injected with LPS 0.8 mg/kg once to induce Alzheimer's disease. Five of them were treated starting 1st day after LPS intraperitoneal injection with PCT 2.5 mg/kg or 100 mg/kg/day GT, MP, SBS, or SBL for 6 days. The brains of all the animals in each group were harvested on the 7th day and preserved until amyloid beta 42 concentration was assayed. Statistical analysis was carried out using unpaired *t*-test to compare every two groups. Each value represents mean ± standard error of mean. *Significantly different from normal at *P* < 0.05. **Significantly different from normal at *P* < 0.01. ***Significantly different from normal at *P* < 0.001. ^+^Significantly different from LPS at *P* < 0.05. ^++^Significantly different from LPS at *P* < 0.01. ^+++^Significantly different from LPS at *P* < 0.001. LPS: Lipopolysaccharide. GT: green tea extract of* Camellia sinensis.* PCT: piceatannol. SBS:* Schotia brachypetala* leaves. SBL:* Schotia brachypetala* stalk. MP:* Markhamia platycalyx*. DMSO: dimethyl sulfoxide. Pg/mL: picogram/milliliter. A*β*: amyloid beta. ELISA: enzyme linked immunosorbent assay. i.p.: intraperitoneal injection.

The mean amyloid beta 42 concentration was significantly reduced in animals that were treated with* Camellia sinensis* (100 mg/kg/day), Piceatannol (2.5 mg/kg/day),* Markhamia platycalyx *(100 mg/kg/day), and* Schotia brachypetala* leaves (100 mg/kg/day) and* Schotia brachypetala* stalk (100 mg/kg/day) for 6 days by 72.6%, 73.7%, 75.1%, 76.2%, and 72.8%, respectively, when compared to LPS group (Figure 4).

## 4. Discussion

Hyphenated HPLC-MS technique is an important method used for identifying complex mixtures, especially the phenolics in the crude extracts or its fraction found in the plant, either by using standard compounds (cochromatography) or by comparing mass spectrum obtained with the literature (tentative identification) [16]. In this part of the study, aqueous alcohol extract of* Schotia brachypetala *was subjected to HPLC-ESI-MS analysis aiming at developing a robust LC-ESI-MS^n^ method in the first instance for the identification of major compounds within this extract (Figure 1).

The obtained data (Table 1) was interpreted as follows.

The negative ion mode LC-ESI-MS^n^ showed major peaks (Sb9 and Sb10) which were previously identified in [16] as quercetin hexose gallic acid and its sugar isomer (Sb9 and Sb10) fragments showed deprotonated molecule [M-H]^−^ *m/z* 615, the fragment peak ion corresponding to the deprotonated quercetin hexoside (*m/z* 463), and deprotonated quercetin molecule at (*m/z* 301) [16]; the appearance of two peaks having the same molecular ion and fragmentation pattern assures the presence of the same compound having two different (hexose) isomers; the identification of the sugar moiety could be further confirmed using NMR spectroscopical analysis [30].

Compound (Sb8) showed the product ion spectrum of quercetin-3-*O*-glucuronide known as miquelianin with deprotonated molecule (*m/z* 477) and MS^2^ at* m/z* 301; a difference of 176 indicates glucuronic acid moiety, besides the MS^3^ at* m/z* 151 of ring A in the quercetin aglycone, proving that it is quercetin aglycone and not ellagic acid that is associated with these compounds [15].

Furthermore, the deprotonated ion of (Sb14) showed fragment at (*m/z*) 447, identified as quercetin 3-*O-*rhamnoside similar to what was reported previously [31]. The result of hemolytic cleavage of the* O*-glycosidic bond renders a radical aglycone, (*m/z *301) the ion referring to quercetin ion. The fragment ion at* m/z* 301 is formed by loss of the glucose or galactose moiety from the glycosides. No ions characteristics of the sugar part were observed in the negative ion mode [32].

Compound (Sb13) showed molecular ion peak at* m/z* 599 with fragments* m/z* 463 and* m/z* 300. The difference between ions at* m/z* 599 of 463 and 300 revealed the loss of two consecutive ions with 162 and 136 mass units, respectively, which is characteristic to hexose sugar and protocatechuic acid. Then this compound could be identified as quercetin hexose-protocatechuic acid isomer [17]. In addition, the two major peaks (Sb3 and Sb5) having the same deprotonated ions ([M-H]^−^) at* m/z* 577 identified tentatively as procyanidin isomer gave [M-H-152]^−^ fragments ions at* m/z* 425 from Retro-Diels-Alder (RDA) rearrangement of the heterocyclic ring and at* m/z* 407 ([M-H-170]^−^) from the rearrangement of the heterocyclic ring and loss of H_2_O [14].

Consequently, the identification of these two peaks (Sb3 & Sb5) gave further evidence to presence of the major compound (Sb6) recognized as procyanidin dimer gallate having its major molecular ion at* m/z *577 [3, 14]. Additionally, the ESI-MS-MS spectra of compound (Sb6) with [M-H]^−^ at* m/z *729 showed the fragment ion at* m/z *577; this could be attributed to the ion arising from the loss of galloyl moiety from the procyanidin dimer having a mass unit as [galloyl-OH]^−^. Therefore, it is identified as procyanidin dimer gallate [3].

Moreover, LC-MS-MS method was also used to distinguish between conjugates of quercetin and ellagic acid since their aglycones produce identical molecular ions on fragmentation, both giving the same base peak* m/z *301 [18]. On MS/MS analyses, the quercetin* m/z* 301 ion further fragments to form characteristic* m/z* 179 and 151 ions whereas the equivalent ellagic acid* m/z* 301 ion yields ions at* m/z* 257 and 229. The use of LC-MS-MS methods is therefore useful to differentiate between ellagic acid and quercetin aglycones; thus the* m/z* 257 and 229 ions in the MS-MS analysis showed that an ellagic acid moiety, and not quercetin, was associated with compound shown in peak (Sb15). Compound (Sb1) showed molecular ion peak at (*m/z*) 253, identified as daidzein [13].

In this study, LPS injected at a dose of 0.8 mg/kg resulted in significant changes in mice working memory. This was proven by Y maze where the percentage spontaneous alternations significantly decreased compared to normal mice. These results are in accordance with a study which showed that the injection of LPS had a disruptive effect on the ability of mice to learn the Y maze [33]. Using the Morris water maze task, LPS-treated mice took a longer time to reach the hidden platform than normal mice. In addition, injection of LPS decreased the percent of correct choices in the Y maze test [34]. LPS also successfully induced memory impairment in the Y maze test, neuroinflammatory responses, and oxidative stress such as increases in mRNA levels of interleukin (IL)-1*β* and IL-6, heme oxygenase-1, microglial activation, and iNOS activity in hippocampus [28]. Neuroinflammation and oxidative stress are significant components of the pathogeneses of AD. Therefore, these changes are successful in inducing learning and memory impairment in rats. In this study, intraperitoneal injection of LPS at dose of 0.8 mg/kg significantly reduced discrimination ratio in object recognition test when compared with normal mice. These results are similar to a study where spatial learning and object recognition memory deficits were observed in animals dosed with the increasing LPS dose regime [35]. In another study, there was significant decrease of cognition function 4 days after systemic injection of LPS analyzed by object recognition test [36]. It has been found that amyloid beta 42 is more toxic than amyloid beta 40 [37]. In this study, LPS injected mice showed significantly higher concentrations of amyloid beta 42 compared with normal mice. These results are in agreement with another study in which transgenic APPswe mice's Abeta1-40/42 was 3-fold higher when compared to normal as detected by ELISA, western blots, and immunoprecipitation-mass spectrometry (IP-MS) ProteinChip analysis [38]. In a study done by [39] LPS was able to induce long lasting modifications in behavior and brain protein levels of TNF-*α*. TNF-*α* levels after seven days of LPS exposure were the same as those after 10 months; these results suggest that LPS reaches its peak effect after 7 days of its injection after which it reaches a plateau.

Another study in harmony with this one showed a decrease in preference of the novel object after LPS injection [28], in which intraperitoneal injection of green tea extract resulted in significantly higher discrimination ratio in object recognition test compared with nontreated LPS mice. In this study, treatment of LPS injected mice with green tea phenolic rich extract significantly increased their spontaneous alternations percentage in the Y maze test compared to nontreated mice. This is in accordance with a study in which long-term administration of green tea catechins to mice also suppressed cognitive dysfunction by assessment of working memory in the Y maze [40]. Administration of green tea catechins improved spatial cognition learning ability in rats assessed by partially baited 8-arm radial maze and was thought to be due to green tea catechins involved in protecting against neuronal degenerative stress and in the accumulation of lipid peroxides (LPO) and reactive oxygen species (ROS). Tea is rich in polyphenols contained in the leaves and stems of the tea plant. EGCG, the major and most active component of green tea catechins, acts as an antioxidant in the biological system and is rapidly absorbed and distributed mainly into the mucous membranes of the small intestine and the liver; more interestingly, it can cross the blood brain barrier. It was found that oxidative stress-induced neuronal apoptosis is prevented by EGCG treatment of neuronal cells [41]. The working memory, tested using Y maze, was improved in mice fed a high-fat diet containing green tea catechin [42]. EGCG treatment significantly improved results of the Y maze in streptozotocin diabetic rats [43]. In this study, intraperitoneal injections of green tea extract caused significantly decreased levels of amyloid beta 42 compared with LPS mice. This may be due to the ability of EGCG to increase alpha secretase activity (the nonamyloidogenic pathway of APP processing) as discovered in a transgenic Alzheimer's disease mouse model [44].

In this study, piceatannol administered to mice produced significantly higher percentage spontaneous alternations in the Y maze compared to nontreated mice. Piceatannol (trans-3,4,3′,5′-tetrahydroxystilbene) is a naturally occurring hydroxylated analogue of resveratrol. Tetrahydroxy stilbene glucoside treatment in aged rats showed a remarkable improvement in their learning and memory function as noted by a marked decrease in the escape latency time, an increase in the time spent in the target quadrant, and an increase in the times of crossing the quadrant where the platform was previously placed in the Morris water maze test [45]. A study investigating the possible protective effects of piceatannol on amyloid beta-induced PC12 neuronal cell death found that piceatannol exerted much stronger protective effects than resveratrol did. Piceatannol treatment attenuated the intracellular accumulation of ROS induced by treatment of PC12 cells with amyloid beta, inhibited amyloid beta-induced apoptotic features including internucleosomal DNA fragmentation, nucleus condensation, cleavage of poly(ADP-ribose) polymerase (PARP), and activation of caspase-3. These results suggest that piceatannol blocks amyloid beta-induced accumulation of ROS, thereby protecting PC12 cells from oxidative stress [46]. In this study, intraperitoneal injection of piceatannol resulted in significantly higher discrimination ratio in the object recognition test compared to LPS nontreated mice. In another study in which the effect of piceatannol to stop apoptosis of cells was investigated (as apoptosis plays an important role in Alzheimer's disease), piceatannol inhibited 4 hydroxynonenal induced apoptosis of PC12 cells. It inhibited the phosphorylation of c-Jun N-terminal kinase, a key regulator of HNE-induced PC12 cell death [47]. This can explain why piceatannol treated mice had greater discrimination ratio. In this study, intraperitoneal injections of piceatannol were able to significantly reduce amyloid beta 42 compared with normal mice. Piceatannol decreased amyloid beta 1–42 induced fluorescence in a TH-t fluorescence assay [7]. It is able to inhibit the toxic effect of amyloid beta on cells which causes neuronal cell death [46].

Two phenolic rich extracts of* Schotia brachypetala* and* Markhamia platycalyx* were investigated for the first time in this study. Their administration to LPS injected mice resulted in higher percentage spontaneous alternations compared to nontreated mice in the Y maze. Polyphenols influence mental health and cognition, namely, via energy metabolism and modification of signaling pathways and gene expression involved in the ability of a neuron to strengthen and change synaptic connections. In addition to their antioxidant and anti-inflammatory activity, polyphenols have been coupled with the increased expression of BDNF (a neurotrophin known for its influence on the maintenance, survival, growth, and differentiation of neurons), assisting in the reversal of neuronal atrophy and behavior deficits [48]. Polyphenol rich foods: spinach, strawberry, and blueberry extracts were effective in reversing cognitive deficits in Morris water maze performance function among aged rats. Effects of blueberries on both motor and cognitive behavior might involve actions other than antioxidant or antiinflammatory activities. Several studies showed that at least some of these actions may include alterations in signaling [49]. Pomegranate containing high levels of polyphenols including ellagic acids administered to transgenic mice with a mutated APP gene resulted in improved performance in cued and spatial learning tasks compared to sugar water controls. Grape juice containing high levels of flavonoids when administered to rats improved cognitive performance in Morris water maze [50]. Silibinin, a polyphenol belonging to the flavonoid class, was able to overcome the impairment caused by injected amyloid beta. A*β*25-35-injected mice showed significantly reduced spontaneous alternation behaviour compared with vehicle-injected mice. Treatment with silibinin dose-dependently attenuated the impairment of spontaneous alternation behaviour in A*β*25-35-injected mice [51]. In this study, mice receiving phenolic rich injections of* Markhamia platycalyx* and* Schotia brachypetala* extracts produced significantly higher discrimination ratio in the object recognition tests compared to nontreated mice. In a model of amyloid beta deposition, the polyphenol oleuropein aglycone improved cognition assayed by object recognition test [52]. In this study, intraperitoneal injections of phenolic extracts of* Markhamia platycalyx* and* Schotia brachypetala* were able to significantly reduce amyloid beta 42 compared to LPS mice. From the compounds that were identified in* Schotia brachypetala* in this study, extracts of flavan-3-ols procyanidin B isomers (Sb3, Sb5) have been shown to be potentially useful in the treatment of degenerative diseases associated with oxidative stress as Alzheimer's disease. This was proven in a study in which they were able to prevent lipid and protein oxidative damage in the cerebral cortex, cerebellum, and hippocampus tissues of rats [53]. Moreover, procyanidins significantly suppressed A*β*42 aggregation and were able to break apart A*β*42 aggregates in a dose-dependent manner [54]. From compounds identified in* Schotia brachypetala* in this study, daidzein (Sb1), is an isoflavone which has been associated with a decreased risk of hormones dependent neurodegenerative diseases [13]. One of the hypotheses of Alzheimer's disease involves oxidative stress. The antioxidant activity of procyanidin dimers (Sb6) (identified from* Schotia brachypetala*) containing the interflavan bond C4–C8 and C4–C6 was very significant, suggesting that interflavan linkage contributes importantly to the antioxidant activity of procyanidin dimers [55]. Ellagic acid (Sb15) (also identified from* Schotia brachypetala*) can have therapeutic potential in Alzheimer's disease as it was able to significantly reduce Abeta 42 induced neurotoxicity towards SH-SY5Y cells (human derived cell line) [56]. Quercetin 3-*O*-glucuronide (identified from* Schotia brachypetala*) greatly reduced the generation of *β*-amyloid (A*β*) peptides by primary neuron cultures generated from the Tg2576 AD mouse model. Quercetin 3-*O*-glucuronide (Sb8) is also capable of interfering with the initial protein-protein interaction of A*β*(1–40) and A*β*(1–42) that is necessary for the formation of neurotoxic oligomeric A*β* species. Moreover, quercetin 3-*O*-glucuronide treatment, compared to vehicle-control treatment, significantly improved AD-type deficits in hippocampal formation basal synaptic transmission and long-term potentiation, possibly through mechanisms involving the activation of the c-Jun N-terminal kinases and the mitogen-activated protein kinase signaling pathways. Therefore, quercetin 3-*O*-glucuronide is an effective intervention for AD [57]. Pretreatment of primary hippocampal cultures with quercetin significantly attenuated Abeta (1–42) induced cytotoxicity, protein oxidation, lipid peroxidation, and apoptosis. Therefore, quercetin greatly protects neuronal cells from oxidative stress-induced neurotoxicity [58]. In another study, phenolic glycosides from* Markhamia stipulata *were isolated, mainly being verbascosides [10]. Verbascosides are phenylpropanoids that have cytoprotective action connected to their antioxidant and chelating capacity. They were found protective against 1-methyl 4 phenylpyridine ion induced neurotoxicity in cultured neurons. They attenuated neuronal apoptosis, caspase 3 activation, and the collapse of mitochondrial membrane potential. This data is proof that* Markhamia* genus can have a therapeutic potential for treating oxidative stress related neurodegenerative disease [11] and supports the positive results in this study.

Finally, binding and autoradiographic studies revealed the existence of specific polyphenols binding sites in the rat brain, in particular in the choroid plexus. Structure-activity data support the hypothesis that these specific binding sites may be responsible for the neuroprotective actions of polyphenols [7].

## 5. Conclusion

The results of this study provide evidence that the phenolic rich extracts of* Schotia brachypetala, Camellia sinensis, Markhamia platycalyx*, and piceatannol have high potential to be anti-Alzheimer's disease drug leads. The pharmacological screening indicated in the Y maze test that all the drugs had significantly higher spontaneous alternation percentage than the nontreated LPS injected mice. In the object recognition test, all the drugs had significantly higher discrimination ratio than the nontreated LPS injected mice as well. The drugs were also able to significantly decrease the amyloid beta 42 burden in mice that were treated compared to nontreated LPS injected mice as detected by ELISA. The identification of compounds procyanidin isomers, daidzein, naringin, procyanidin dimer gallate, quercetin 3-*O*-glucoronide, quercetin hexose gallic acid, quercetin hexose-protocatechuic acid, quercetin 3-*O*-rhamnoside, and ellagic acid seems to be limited within the complexity of the extract, but this study is directing towards the major compounds in the extract and to be correlated with the biological activity; the information provided by our study will aid in the evaluation of the biological importance of* Schotia brachypetala* consumption on human health.

Further work would involve the identification and isolation of the identified compounds to further enrich the literature concerning such a plant by other tools such as LC-UV and nuclear magnetic resonance (NMR). Further work would also involve the investigation of the mechanisms of actions of these compounds and physiochemical identification of polyphenolic compounds in* Markhamia platycalyx*. Pharmacological screening using other methods such as western blot can further confirm the relative pharmacological activity of the extracts.

## Figures and Tables

**Table 1:** Peak assignment, mass spectrometry detection parameters and tentative identification of compounds in aqueous alcohol stalks extract of *Schotia brachypetala* by LC-ESI(-ve)-MS.

Peak number	Identified compounds	*t*_*R*_ (min)	[M-H]^−^ (*m*/*z*)	Fragment ions (*m*/*z*)	Peak area %	References
Sb1	Daidzein	1.76	253	253	1.3	[13]
Sb2	Unidentified	12.2	495	253	2.5	
Sb3	Procyanidin isomer B1/B2	15.79	577	425, 407	6.5	[14]
Sb4	Naringin	18.76	579	459, 271	5.2	[14]
Sb5	Procyanidin isomer B1/B2	23.77	577	425, 407	4.8	[14]
Sb6	Procyanidin dimer gallate	29.79	729	577	6.2	[3]
Sb7	Unidentified	38.84	497	441, 253	3.2	
Sb8	Quercetin 3-*O*-glucoronide	42.07	477	301, 151	5.6	[15]
Sb9	Quercetin hexose gallic acid	46.82	615	463, 301	15.5	[16]
Sb10	Quercetin hexose gallic acid	47.63	615	463, 301	10	[16]
Sb11	Unidentified	48.82	463	298, 253	8.3	
Sb12	Unidentified	50.8	495	239, 220	6.6	
Sb13	Quercetin hexose-protocatechuic acid	52	599	463, 300	7.2	[17]
Sb14	Quercetin 3-*O*-rhamnoside	57.04	447	301	9.2	[18]
Sb15	Ellagic acid	64.64	301	257, 229	4.1	[18]

## Abbreviations

AD: Alzheimer's disease
CNS: Central nervous system
Ach: Acetylcholine
AchE: Acetylcholinesterase
DMSO: Dimethylsulfoxide
LPS: Lipopolysaccharide
LC-HRESI-MS^n^: Liquid chromatography high resolution electrospray ionization mass spectrometry.
